# Interplay of KIR2DL5, nitric oxide, and tobacco smoking in predisposition to bladder cancer

**DOI:** 10.3389/fcell.2025.1632101

**Published:** 2025-09-04

**Authors:** Inmaculada Ruiz-Lorente, Lourdes Gimeno, Alicia López-Abad, Pedro López Cubillana, Tomás Fernández Aparicio, Lucas Jesús Asensio Egea, Juan Moreno Avilés, Gloria Doñate Iñiguez, Pablo Luis Guzmán Martínez-Valls, Gerardo Server, Belén Ferri, José Antonio Campillo, Francisco Galindo, Francisco Boix, María Victoria Martínez-Sánchez, María Dolores Martínez-Hernández, Alfredo Minguela

**Affiliations:** ^1^ Immunology Service, Virgen de la Arrixaca University Clinical Hospital (HCUVA), Biomedical Research Institute of Murcia (IMIB), Murcia, Spain; ^2^ Human Anatomy Department, Universidad de Murcia and Campus Mare Nostrum, Murcia, Spain; ^3^ Urology Service, Virgen de la Arrixaca University Clinical Hospital (HCUVA), Biomedical Research Institute of Murcia (IMIB), Murcia, Spain; ^4^ Urology Service, Morales Meseguer Hospital, Murcia, Spain; ^5^ Urology Service, De la Vega Lorenzo Guirao Hospital, Murcia, Spain; ^6^ Urology Service, Santa Lucia Hospital, Murcia, Spain; ^7^ Urology Service, Los Arcos Hospital, Murcia, Spain; ^8^ Urology Service, Reina Sofía Hospital, Murcia, Spain; ^9^ Pathology Service, Virgen de la Arrixaca University Clinical Hospital (HCUVA), Biomedical Research Institute of Murcia (IMIB), Murcia, Spain; ^10^ Departament of Inorganic and organic Chemistry, Universitat Jaume I, Castellón, Spain; ^11^ Centro de Transfusión de la Comunidad Valenciana, Valencia, Spain

**Keywords:** bladder cancer predisposition, KIR2DL5, immune response, nitric oxide, tobacco smoking

## Abstract

**Introduction:**

Tobacco smoking is the most significant risk factor for bladder cancer (BC), followed by other environmental and dietary exposures. However, major genetic determinants remain unidentified. The objective of this work was to investigate the potential association of killer-cell immunoglobulin-like receptor 2DL5 (KIR2DL5) with BC risk, its interaction with tobacco smoking, and the underlying immune mechanisms.

**Methods:**

This case-control study analyzed KIR genotype in patients with BC (n = 325), as well as in healthy controls (HC, n = 925) and patients with other cancers (n = 862) as control groups. Immune assays assessed proliferation, cytotoxicity, cytokine, and intracellular nitric oxide (icNO) production by NK and T cells after anti-CD3/CD28 or *Bacillus* Calmette-Guérin (BCG) stimulation in 24 donors stratified by KIR2DL5 genotype. Multivariate logistic regression was used to evaluate BC predisposition.

**Results:**

The frequency of KIR2DL5 was higher in BC patients than in HC (64.6% vs. 53.6%, p = 0.004). Linear regression analysis revealed that, independent of other aKIRs within the B haplotype, KIR2DL5 was associated with BC susceptibility (HR = −1.167, p = 0.050), alongside other significant factors such as sex (HR = −1.465, p < 0.001), age (HR = −0.181, p < 0.001), and tobacco smoking (HR = −2.454, p < 0.001). The frequency of KIR2DL5 was higher among non-smokers compared to smokers in both healthy controls (61.4% vs. 44.6%, p < 0.05) and BC patients (72.9% vs. 60.8%, p < 0.05). Among non-smoking BC patients, KIR2DL5 was more frequently observed in small-sized (<3 cm), solid-pattern, non-muscle-invasive BC cases. Immune profiling revealed that KIR2DL5 was associated with increased icNO production by NK and T cells but showed no association with proliferation, cytokine secretion, or cytotoxicity.

**Discussion:**

KIR2DL5 is independently associated with BC, regardless of age, sex, or tobacco smoking status. While the immunological mechanisms remain unclear, enhanced nitric oxide production by immune effector cells may play a role in this association.

## 1 Introduction

KIR2DL5 (CD158f) is the most recently identified functional inhibitory member of the killer-cell immunoglobulin-like receptor (KIR) family. Unlike its homolog KIR2DL4, which is ubiquitously expressed in all individuals, KIR2DL5 exhibits copy number variation and is present in only a subset of the population (Vilches et al., 2000). The human genome contains two paralogous genes encoding this receptor: KIR2DL5A and KIR2DL5B, which display allelic polymorphism and population-specific distributions (Gómez-Lozano et al., 2002). KIR2DL5 is clonally expressed on subsets of natural killer (NK) cells (particularly CD56^dim^ populations) and T lymphocytes (Cisneros et al., 2012). Its inhibitory function is mediated by a long cytoplasmic tail containing two immunoreceptor tyrosine-based inhibitory motifs (ITIMs). Upon receptor engagement, phosphorylated ITIMs recruit Src homology region 2 (SH2)-containing phosphatases SHP-1 and SHP-2, with a predominant role for SHP-2 in suppressing NK cell activation. This signaling mechanism distinguishes KIR2DL5 from classical inhibitory KIRs (iKIR) that primarily utilize SHP-1-dependent pathways (Yusa et al., 2002; Yusa and Campbell, 2003; Ren et al., 2022).

Both the centromeric and telomeric KIR2DL5 loci are followed by paralogs of a duplicated KIR2DS3S5 genes, each encoding different alleles of the activating KIRs (aKIRs) KIR2DS3 and KIR2DS5 (Ordóñez et al., 2008). Additionally, KIR2DL5 is strongly associated, within the B haplotype, with the aKIR genes KIR2DS1 and KIR3DS1. The complex polymorphism and strong linkage disequilibrium with neighboring KIR genes complicate the evaluation of the individual role of KIR2DL5 as either a risk or protective factor in various pathologies. Nonetheless, a higher frequency of aKIRs or B haplotypes has been associated with susceptibility to gastric cancer (Hernandez et al., 2018), non-Hodgkin lymphoma (Pamuk et al., 2015), head and neck squamous cell carcinoma (Barani et al., 2018), childhood acute lymphoblastic leukemia (Misra et al., 2016), poorer prognosis in non-Hodgkin lymphoma (Pamuk et al., 2015), higher frequency of minimal residual disease after treatment in childhood acute lymphoblastic leukemia (Sullivan et al., 2014), and significantly worse survival in patients with hematological malignancies after hematopoietic stem cell transplantation from unrelated donors (Nowak et al., 2015). In bladder cancer (BC), KIR2DL5 is associated with a higher risk and poorer prognosis. High-risk patients identified with the KIR2DL5^+^/HLA-C*16^+^ genotype showed significantly shorter progression-free and overall survival compared to other genotypes (Guillamón et al., 2021).

The identification of the poliovirus receptor (PVR, CD155) as a ligand for KIR2DL5 in 2019 has opened new avenues of research in cancer immunotherapy (Husain et al., 2019), particularly in the context of BC. PVR is significantly upregulated in muscle-invasive BC (MIBC) compared to matched normal urothelium (Zhang et al., 2020). KIR2DL5 functions as an inhibitory receptor by binding to PVR on tumor cells, promoting the formation of inhibitory synapses and suppressing NK cell cytotoxicity (Ren et al., 2022). Increased PVR expression has been associated with a higher risk of recurrence in patients with non-muscle invasive BC (NMIBC), indicating that PVR could serve as an important biomarker for assessing recurrence risk in this population (Al-Zubi et al., 2022). In fact, blocking the KIR2DL5/PVR interaction with monoclonal antibodies has been shown to enhance NK cell-mediated cytotoxicity against PVR^+^ tumors and reduce tumor growth, thereby improving overall survival in humanized tumor models (Ren et al., 2022). This suggests that such a strategy could be effective in improving NK cell function in cancer immunotherapy. Nonetheless, PVR is also a noncompetitive ligand for the (co)stimulatory receptor DNAM-1 (CD226) as well as the inhibitory receptors TIGIT and CD96. Although TIGIT binds to PVR with higher affinity than CD226, the integrated signals generated by these complex interactions ultimately determine the outcome of antitumor responses (Yeo et al., 2021). Thus, co-targeting the interactions of both KIR2DL5 and TIGIT with PVR holds great potential as a therapeutic approach to enhance NK cell-mediated antitumor immunity in BC (Kučan Brlić et al., 2019).

Smoking tobacco is the single most important known risk factor for BC, contributing to up to 50% of bladder tumors (Freedman, 2011; Halaseh et al., 2022). More than 5,000 chemicals, including 62 carcinogens, have been detected in tobacco, which may contribute to tumorigenesis through the activation of oncogenes, inhibition of tumor suppressor genes, induction of genetic and epigenetic changes, and alteration of growth pathways, angiogenesis, and metastasis (Nooshinfar et al., 2017). However, chronic smoking can also impair endothelial function by decreasing the formation of nitric oxide (NO) and increasing its degradation (Toda and Toda, 2010). NO signaling can inhibit hypoxia-induced tumor cell invasiveness, metastatic capacity, and resistance to chemotherapeutic agents (Siemens et al., 2008), therefore, NO inhibition could represent an additional tumorigenic mechanism of tobacco. Nonetheless, the role of NO in T and NK cell immune functions remains controversial (Bogdan, 2001).

In this study, we investigated the relationship between KIR2DL5, as a potential new immunotherapeutic target in BC, and the antitumor functionality of NK and T lymphocytes, smoking habits, and patient survival. Our data reveal a clear association of KIR2DL5 with BC susceptibility, in which both NO and tobacco smoking may be involved.

## 2 Materials and methods

### 2.1 Samples and study groups

This prospective, observational, case-control study included 925 healthy Caucasian (HC) volunteers as the control group and 1,190 consecutive cancer patients as the experimental group. The cancer cohort comprised patients with BC (n = 328), melanoma (n = 310), plasma cell neoplasms (n = 335), pediatric acute leukemia (n = 128), and ovarian cancer (n = 89). The BC population consisted of two distinct cohorts, each recruited during separate time frames as part of different research projects (https://orcid.org/0000-0003-2472-5893): Series 1 (2014–2016, n = 136) and Series 2 (2019–2022, n = 192). BC tumors were classified according to the WHO Classification of Tumours of the Urinary System and Male Genital Organs (Moch et al., 2016) into: 1) noninvasive urothelial neoplasms (NIUN), including urothelial carcinoma *in situ* (CIS) and low- and high-grade papillary carcinomas (Ta); and 2) infiltrating urothelial carcinoma (IUC), including NMIBC T1-stage and MIBC T2, T3, and T4 stages. Progression in NIUN was defined as local recurrence with a higher grade or stage, and in IUC as local recurrence with a higher stage and/or development of metastatic disease. Treatment and management were at the discretion of the urologists, based on patient condition and tumor histology. The study was approved by the Research Ethics Committee (Institutional Review Board IRB-00005712). Written informed consent was obtained from all patients and controls in accordance with the Declaration of Helsinki.

Peripheral blood samples anticoagulated with EDTA (for HLA genotyping and NK cell receptor expression analysis by flow cytometry) were obtained at diagnosis prior to any treatment. Fresh sodium heparin blood samples were collected from selected healthy donors for proliferation and cytotoxicity assays, as well as for intracellular nitric oxide (icNO) and cytokine production assays.

### 2.2 KIR genotyping

KIR genotyping was conducted on DNA extracted from peripheral blood using the QIAamp DNA Blood Mini Kit (QIAGEN, Hilden, Germany) and Lifecodes KIR-SSO typing kits (Immucor Transplant Diagnostic, Stamford, CT, United States), following established protocols (Guillamón et al., 2018; Gimeno et al., 2021). The analysis identified inhibitory KIRs (2DL1–2DL3, 2DL5, 3DL1–3DL3), activating KIRs (2DS1–2DS5, 3DS1), and KIR2DL4, which has both inhibitory and activating functions (Moretta and Moretta, 2004). The method used could not distinguish between the telomeric (KIR2DL5A) and centromeric (KIR2DL5B) forms. Genotypes were classified as AA if they contained only the canonical A-haplotype genes (KIR3DL3, KIR2DL3, KIR2DL1, KIR2DL4, KIR3DL1, KIR2DS4, and KIR3DL2) (Hsu et al., 2002). Any genotype containing additional KIR genes was designated as Bx.

### 2.3 Expression of NK cell receptors in peripheral blood lymphocytes

The expression of CD226 (DNAM-1), NKG2A, TIGIT, and KIR receptors (KIR2DL1, 2DS1, 2DL2/S2, 2DL3, and 3DL1) was simultaneously assessed on both CD56^bright^ and CD56^dim^ NK cells, as well as on CD3^+^CD4^+^ and CD3^+^CD8^+^ T cells, using LSR-II or Lyric flow cytometers and DIVA software (BD), according to previously published protocols (Guillamón et al., 2018; Guillamón et al., 2021). Peripheral blood samples were stained with the following monoclonal antibodies: CD158a-FITC (143211, R&D Systems Inc., recognizing KIR2DL1), CD158a/h-PC7 (EB6B, Beckman Coulter, recognizing both KIR2DL1 and 2DS1), CD158b2 (180701, R&D Systems Inc., KIR2DL3), CD226-PE (11A8, Biolegend), CD158e1 (DX9, R&D Systems, KIR3DL1), CD16-AlexaFluor700 (3G8, BD), CD8-APC-Cy7 (SK1, BD), TIGIT-BV421 (741182, BD), CD3-BV510 (UCHT1, BD), CD4-BV605 (RPA-T4, BD), CD56-BV711 (NCAM16.2, BD), and CD159a-BV786 (131411, BD). Cells were incubated with antibodies for 10 min at room temperature in the dark, followed by red blood cell lysis and washing before acquisition.

### 2.4 *In vitro* functional assays

To assess the impact of KIR2DL5 on T and NK cell function, we evaluated the proliferation, cytotoxicity against tumor cell lines (K562, J82, and T24), and the production of icNO and cytokines by peripheral blood mononuclear cells (PBMCs). PBMCs were isolated using Ficoll density gradients from sodium heparin-anticoagulated blood samples obtained from 24 healthy donors (12 KIR2DL5-negative and 12 KIR2DL5-positive). PBMCs were stained with 0.05 µM carboxyfluorescein succinimidyl ester (CFSE; Thermo Fisher Scientific, Waltham, MA) and stimulated *in vitro* either with ImmunoCult™ Human CD3/CD28 T cell activator (Stemcell Technologies, Vancouver, Canada), according to the manufacturer’s instructions, or with BCG (Danish 1331, AJVaccines, Copenhagen) at a 1:1 colony-forming unit (CFU) to PBMC ratio, as previously described (Ruiz-Lorente et al., 2025). CFSE-labeled cells (1 × 10^6 per well) were cultured in 24-well flat-bottom plates (five replicates per condition) at 37 °C in a 5% CO_2_ incubator. At 72 h, supernatants from one well per sample were collected and stored at −80 °C for subsequent cytokine analysis by Luminex. At 120 h, cells from one well per sample were harvested for icNO detection by FACS-Lyric flow cytometry. After 144 h, the remaining cells were used for cytotoxicity assays or stained for proliferation analysis using a Northern Light (NL) flow cytometer (Cytek, Amsterdam, Netherlands).

### 2.5 Cytokine production

Culture supernatants were analyzed using a ProcartaPlex Human Immune Monitoring 12-Plex Panel (IL-1β, IL-2, IL-4, IL-5, IL-6, IL-8, IL-10, IL-17A, IL-22, IL-23, IFN-γ, TNF-α, and TGF-β1; Thermo Fisher Scientific, Vienna, Austria) following the manufacturer’s instructions. The analysis was performed on a Luminex 300 system (R&D Systems, Minneapolis, MN, United States) and processed using the ProcartaPlex Analysis App software (Thermo Fisher).

### 2.6 Intracellular NO (icNO)

**FIGURE 1:**
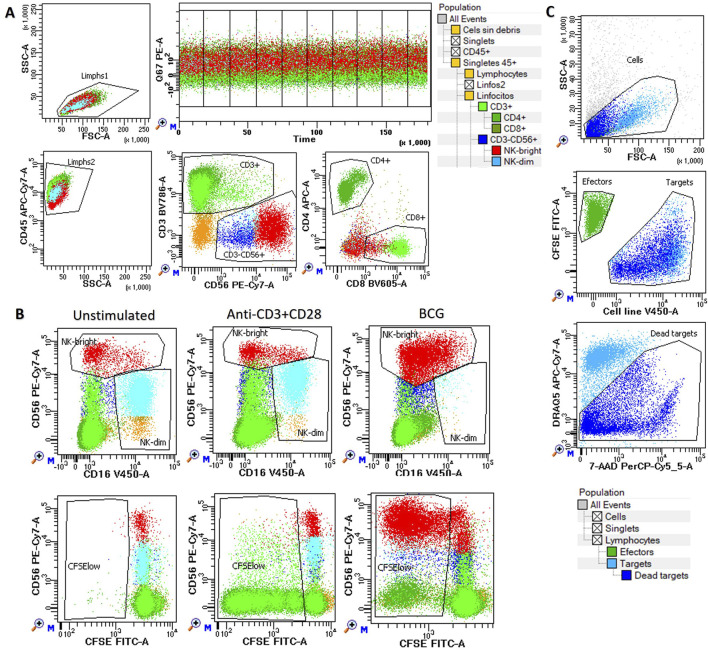
Functional assays of NK and T cells. Peripheral blood mononuclear cells (PBMCs) were cultured in vitro either unstimulated or stimulated with anti-CD3/CD28 or BCG for up to 144 h to assess: **(A)** Intracellular nitric oxide (icNO): Measured at 120 h using mtNOpy in the PE channel (488 nm) in CD4^+^ (dark green), CD8^+^ (light green), NK CD56^dim^ (blue), and NK CD56^bright^ (red) cells. **(B)** Cell proliferation: Assessed at 144 h as the percentage of CFSE-low cells within CD3^+^CD4^+^ (dark green) and CD3^+^CD8^+^ (light green) T lymphocytes, as well as CD56dim (blue) and CD56bright (red) NK cells. **(C)** Cytotoxicity: evaluated against K562, T24, and J82 target cell lines at multiple effector-to-target ratios. Effector PBMCs are shown in green, while target cells are depicted as pale blue for live cells and dark blue for dead cells. In all assays, a hierarchical and logical gating strategy was applied to exclude doublets, debris, and non-viable events, ensuring precise identification of cell subsets and their functional responses.

The analysis was performed as previously described (Muñoz Resta et al., 2021). Briefly, harvested cells were labeled with monoclonal antibodies CD3-BV786 (SK7, Becton-Dickinson, BD), CD4-APC (SK3, BD), CD8-BV605 (SK1, BD), CD45-APC-Cy7 (2D1, BD), CD16-V450 (3G8, BD), and CD56-PE-Cy7 (NCAM16.2, BD) for 10 min at room temperature. The labeled cells were then transferred to flow cytometry tubes containing 1.5 mL of pre-warmed RPMI medium with 10 μM of a pyrylium probe (mtNOpy) and immediately acquired for 30 min at a low flow rate (time recorded). During acquisition, the tubes were incubated in a 37 °C water bath protected from light. The icNO levels were assessed as the mean fluorescence intensity (MFI) of mtNOpy in the PE channel (586/42 nm) excited by the blue laser (488 nm) using a FACSLyric cytometer and DIVA 9.0 software (BD). Photomultiplier voltages were adjusted beforehand using CS&T beads (BD). The gating strategy used to differentiate NK cells, CD4^+^ T cells, and CD8^+^ T cells is illustrated in Figure 1A.

### 2.7 Cell proliferation

Cell proliferation was evaluated in CFSE labeled cells after 6 days of *in vitro* expansion by labeling with TIGIT-BV421 (RUO, BD), CD16-V450 (3G8, BD), CD4-cFV505 (DK3, Palex), CD226-BV605 (11A8, Biolegend), CD8-BV570 (RPA-T8, Biolegend), TIM-3-BV711 (7D3, BD), TCRgd-BV750 (11F2, BD), NKG2A-BV786 (131411, BD), HLA-DR-cFB548 (L2D3, Cytek), NKG2C-PE (REA205, Miltenyi Biotec), CD25-cFBYG610 (BC96, Cytek), CD158bj-PE-Cy5 (GL183, Beckman Culter), KIR3DL1-APC (DX9, R&D Systems Inc.), CD57-cFR668 (HNK1, Cytek), CD38-cFR685 (HIT2, Cytek), CD3-AF700 (UCHT1, BD), NKG2D-APC-H7 (1D11, Biolegend) and CD45-cFR840 (HI30, Cytek) monoclonal antibodies during 15 min at room temperature. Cells were washed with FACSFlow (BD) and acquired using an NL-Flow cytometer (Cytek), then analyzed with Diva software (BD). Proliferation was calculated as the percentage of CFSE-low cells within each cell subset (CD4^+^ and CD8^+^ T cells, CD56^dim^ and CD56^bright^ NK cells), as illustrated in Figure 1B and described previously (Ruiz-Lorente et al., 2025).

### 2.8 Cytotoxic activity

The cytotoxicity of harvested effector cells was assessed against target cell lines stained with CellTrace™ Violet (Thermo Fisher Scientific, Waltham, MA) at effector-to-target ratios of 5:1 and 15:1, performed in triplicate as previously described (Ruiz-Lorente et al., 2025). In parallel, target cells were incubated alone to measure basal cell death. The cells were co-incubated in V-bottom 96-well microplates with a total volume of 150 μL of complete medium for 4 h in a 5% CO2 atmosphere at 37 °C. After incubation, the cell mixtures were washed with PBS containing 1% BSA and stained in the same buffer with 20 µg/mL 7-aminoactinomycin D (7-AAD, Sigma, France) and 0.5 µg/mL DRAQ5 (BD, Canada) for 10 min at 4 °C in the dark. The cells were then washed again and immediately analyzed using a FACSLyric flow cytometer. The mean value from triplicates was used to calculate the percentage of lysis as follows: experimental lysis–spontaneous apoptotic target cells. The gating strategy is illustrated in Figure 1C.

### 2.9 Statistical analysis

Data were collected using Excel 2010 (Microsoft Corporation, Seattle, WA, United States) and analyzed with SPSS version 21.0 (SPSS, Chicago, IL, United States). Categorical variables were analyzed using chi-square tests, while continuous variables were evaluated using analysis of variance (ANOVA) with *post hoc* tests. Kaplan-Meier survival curves and log-rank tests were employed to assess patient survival outcomes, including progression-free survival (PFS) and overall survival (OS). Time-to-event data (progression or death) were calculated in months from the date of diagnosis.

Linear regression analysis was conducted to investigate the impact of multiple parameters on BC susceptibility. Hazard ratios (HRs) and their corresponding 95% confidence intervals (CIs) were estimated. Statistical significance was defined as p < 0.05.

## 3 Results

### 3.1 Clinical, biological and therapeutic characteristics of the study groups

The study included 328 patients with BC, 335 with plasma cell neoplasms, 310 with melanoma, 128 with pediatric acute leukemia, and 89 with ovarian cancer. Among BC patients, 58 cases were classified as NIUN (CIS or Ta), while the remaining cases were IUC, including 150 T1, 99 T2, and 21 T3 or T4 tumors. *Bacillus* Calmette–Guérin therapy was administered to 32 CIS or Ta cases (56.1%), 117 T1 cases (78.5%), and 2 T2 cases (2.0%) (Table 1). The healthy control group consisted of 925 individuals with a mean age of 52 ± 0.7 years, of whom 44.5% were male.

**TABLE 1:** Clinical, biological, and therapeutic characteristics of the study groups.

	N	Sex (% male)	Age, years (mean ± SEM)	Follow-up, months (mean ± SEM)
Type of tumor				
Bladder cancer (BC)	328	85.8	71.1 ± 0.6	42.3 ± 1.8
Plasma cell neoplasm	335	50,6	59.2 ± 2,7	54.9 ± 2.9
Melanoma	310	53.8	60.2 ± 1.8	60.3 ± 2.5
Pediatric leukemia	128	61.8	6.98 ± 0.4	50.8 ± 3.1
Ovarian cancer	89	0	58.7 ± 1.1	18.9 ± 2.3
BC stage				
CIS or Ta	58	86.1	72.9 ± 1.2	60.3 ± 5.1
T1	150	85.2	70.1 ± 0.8	45.4 ± 2.3
T2	99	86.9	71.9 ± 1.1	29.3 ± 2.5
T3 or T4	21	85.7	65.3 ± 2.1	29.4 ± 6.8
BC treatment				
BCG therapy*	151	86.2	70.4 ± 0.8	48.4 ± 2.5
Other therapies	174	85.4	71.7 ± 0.8	37.1 ± 2.4

* BCG, therapy was given to 32 cases CIS, or Ta (56.1%), 117 cases T1 (78.5%), and 2 cases T2 (2.0%).

### 3.2 KIR2DL5 is associated with increased susceptibility to bladder cancer but not with patient outcome

**FIGURE 2:**
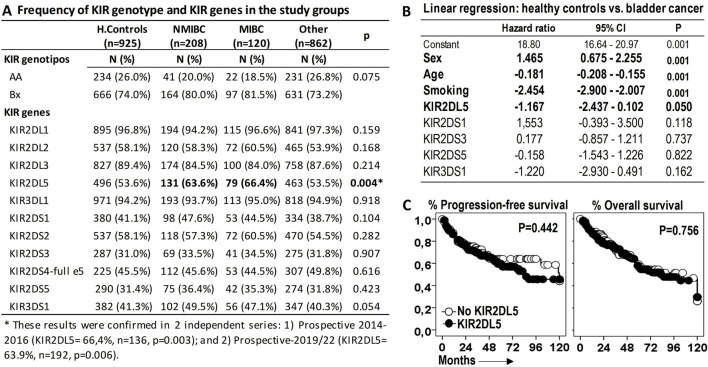
KIR2DL5 is associated with increased susceptibility to bladder cancer (BC) but not with patient outcomes. **(A)** Frequency of KIR genotypes and genes across the study groups. P-values were obtained using chi-square tests. **(B)** Linear multivariate regression analysis comparing BC patients and healthy donors, adjusted for sex, age, and activating KIRs (aKIRs). **(C)** Kaplan-Meier survival curves and Log-rank tests for progression-free survival and overall survival in BC patients stratified by KIR2DL5 genotype.

To investigate the relationship between BC and KIR genotypes or genes, we first analyzed their association. KIR2DL5 emerged as the only KIR receptor significantly associated with BC susceptibility, with frequencies of 63.6% and 66.4% in non-muscle-invasive (NMIBC) and muscle-invasive bladder cancer (MIBC), respectively (p = 0.003), compared to healthy controls (53.6%) and patients with other tumors (53.5%). These findings were validated in two independent prospective BC cohorts: 1) 2014–2016 cohort: KIR2DL5 frequency was 66.4% (n = 136, p = 0.003), and 2) 2019–2022 cohort: KIR2DL5 frequency was 63.9% (n = 192, p = 0.006) (Figure 2A).

The reported frequency of KIR2DL5 in healthy populations from regions near our study area aligns closely with our observations (http://www.allelefrequencies.net/kir6002a.asp): Granada (52.0%, n = 100), Sevilla (54.7%, n = 278), and Valencia (53.7%, n = 1334). Similarly, a series of 458 COVID-19 patients from Valencia reported a frequency of 55.5% (Niño-Ramírez et al., 2024).

KIR2DL5 have a strong association with other aKIRs within the B haplotype. To assess its independent contribution, a linear multivariate regression analysis was performed, revealing that, independent of other aKIRs within the B haplotype, KIR2DL5 was associated with BC susceptibility (HR = −1.167, p = 0.050), alongside other significant factors such as sex (HR = 1.465, p < 0.001), age (HR = −0.181, p < 0.001), and tobacco smoking (HR = −2.454, p < 0.001) (Figure 2B).

However, KIR2DL5 alone was not linked to significant differences in PFS or OS among BC patients (Figure 2C).

### 3.3 KIR2DL5 is associated with an increased frequency of circulating NK cells expressing other B haplotype-associated KIRs

**FIGURE 3:**
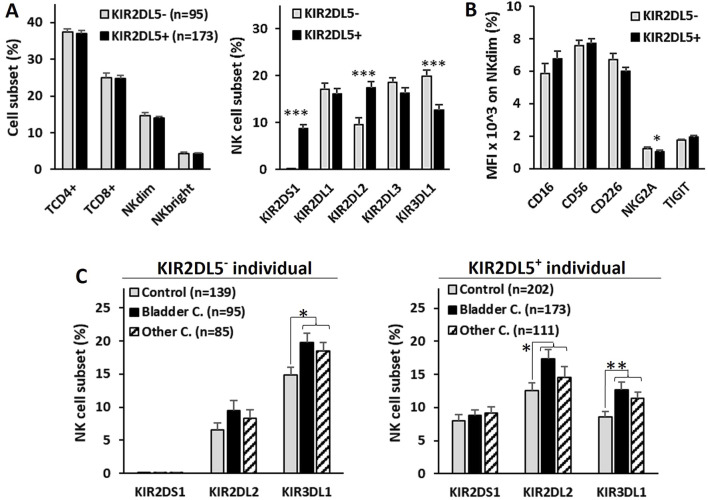
Repertoire of T and NK lymphocyte subsets in peripheral blood and expression of activating and inhibitory NK cell receptors in bladder cancer patients. **(A)** Frequency of CD4^+^ and CD8^+^ T lymphocytes, CD56^dim^ and CD56^bright^ NK cells, and the NK single-KIR^+^ (sKIR) repertoire, stratified by KIR2DL5 genotype. **(B)** Mean fluorescence intensity (MFI) of activating receptors (CD226 and CD16) and inhibitory receptors (TIGIT and NKG2A) on CD56^dim^ and CD56^bright^ NK cells, stratified by KIR2DL5 genotype. **(C)** Frequency of NK cell subsets expressing KIR with significant differences between KIR2DL5-positive and -negative individuals in B, comparing healthy controls and patients with bladder cancer or other cancers. *, p < 0.05, **, p < 0.01 and *** and p < 0.001 based on ANOVA test.

To explore the potential impact of the KIR2DL5 genotype on antitumor effector cells, peripheral blood samples from 268 BC patients at diagnosis were analyzed for NK and T lymphocyte repertoires, as well as the expression of activating receptors (CD226, CD16) and inhibitory receptors (TIGIT, NKG2A) on NK cells (Figure 3). No significant differences in the frequencies of CD4^+^ and CD8^+^ T lymphocytes or CD56^dim^ and CD56^bright^ NK cells were observed between KIR2DL5^+^ and KIR2DL5^−^ patients. However, KIR2DL5^+^ patients exhibited a significantly higher frequency of NK cells expressing KIR2DS1 (8.79% ± 0.58% vs. 0.0% ± 0.0%, p < 0.001) and KIR2DL2 (17.41% ± 1.32% vs. 9.52% ± 1.53%, p < 0.001), alongside a lower frequency of NK cells expressing KIR3DL1 (12.65% ± 1.17% vs. 19.82% ± 1.38%, p < 0.001) compared to KIR2DL5^−^ patients (Figure 3A).

While no differences in the expression of CD226, CD16, or TIGIT were associated with the KIR2DL5 genotype, NKG2A expression was slightly lower in KIR2DL5^+^ patients (1.03% ± 0.1% vs. 1.24% ± 0.09%, p < 0.05) compared to KIR2DL5^−^ patients (Figure 3B).

To investigate whether differences observed in circulating NK cell subsets between KIR2DL5^+^ and KIR2DL5^−^ individuals might play a specific role in the development of BC, the frequencies of these subpopulations were analyzed in healthy controls and patients with BC or other cancers (Figure 3C). Patients with both types of cancer exhibited higher numbers of NK cells expressing KIR2DL2 and KIR3DL1 compared to healthy controls, in both KIR2DL5^+^ individuals (KIR2DL2^+^ NK cells: 9.5% ± 1.5% and 8.3% ± 1.3% vs. 6.5% ± 1.1, p > 0.05; KIR3DL1^+^ NK cells: 19.8% ± 1.4% and 18.5% ± 1.3% vs. 14.9% ± 1.1, p < 0.05) and KIR2DL5^−^ individuals (KIR2DL2^+^ NK cells: 17.4% ± 1.3% and 14.6% ± 1.5% vs. 12.5% ± 1.2, p < 0.05; KIR3DL1^+^ NK cells: 12.6% ± 1.2% and 11.3% ± 1.0% vs. 8.6% ± 0.7, p < 0.01). However, no differences were observed between patients with BC and those with other cancers, suggesting that these NK cell subsets appear to be associated with cancer development in general, but not specifically with BC.

### 3.4 KIR2DL5 genotype is not associated with differential NK cell effector functions *in vitro*

**FIGURE 4:**
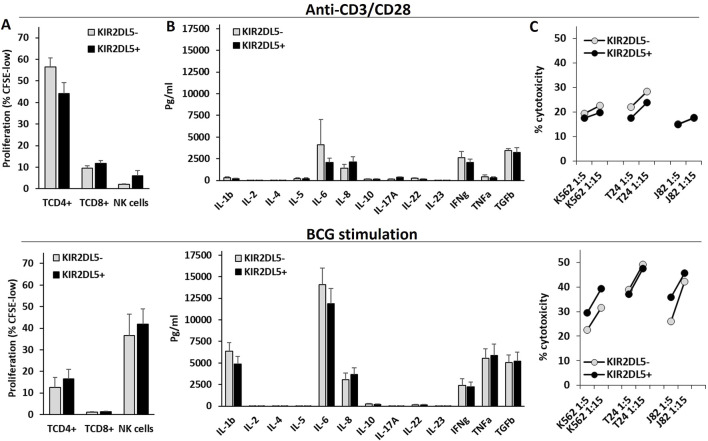
The KIR2DL5 genotype is not associated with differences in NK or T cell proliferation, cytokine production, or cytotoxicity following *in vitro* stimulation with anti-CD3/CD28 or BCG. **(A)** Proliferation (% of CFSE-low) of CD4^+^ and CD8^+^ T lymphocytes and CD56^+^CD3^−^ NK cells; **(B)** Cytokine secretion; **(C)** Cytotoxicity against K562, T24, and J82 cell lines by PBMCs stimulated with anti-CD3/CD28 (upper panels) or BCG (lower panels), stratified by KIR2DL5 genotype.

The effector functions of T and NK cells were assessed in PBMCs from 24 healthy controls stimulated *in vitro* with anti-CD3/CD28 or BCG (Figure 4). Anti-CD3/CD28 primarily induced the proliferation of CD4^+^ T cells, whereas BCG strongly promoted NK cell proliferation and the secretion of IL-1β, IL-6, IFNγ, TNFα, and TGFβ1. However, the KIR2DL5 genotype was not associated with any significant differences in cytokine secretion, T or NK cell proliferation, or the cytotoxic activity of NK cells following stimulation with anti-CD3/CD28 or BCG.

### 3.5 KIR2DL5 is associated with increased intracellular nitric oxide (icNO) production in T and NK cells

**FIGURE 5:**
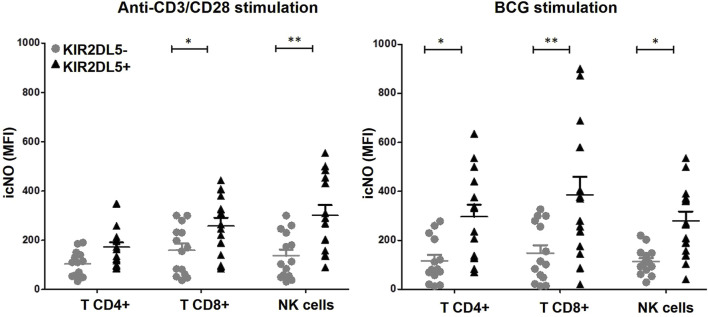
KIR2DL5 is associated with increased intracellular nitric oxide (icNO) production in T and NK cells. Production of icNO was measured in CD4^+^ and CD8^+^ T lymphocytes, as well as CD3^−^CD56^+^ NK cells, from peripheral blood mononuclear cells stimulated with anti-CD3/CD28 or Bacille Calmette–Guérin (BCG), stratified by KIR2DL5 genotype. *p < 0.05 and **p < 0.01, based on Student’s t-test.

Finally, icNO production was evaluated in CD4^+^ and CD8^+^ T lymphocytes, as well as CD3^−^CD56^+^ NK cells, from PBMCs stimulated with anti-CD3/CD28 or BCG, stratified according to the KIR2DL5 genotype (Figure 5). Following anti-CD3/CD28 stimulation, CD4^+^ (171.9 ± 37.9 vs. 103.3 ± 14.9 MFI) and CD8^+^ (257.6 ± 62.3 vs. 159.1 ± 27.6 MFI, p < 0.05) T lymphocytes, along with CD3^−^CD56^+^ NK cells (301.2 ± 58.8 vs. 137.1 ± 17.5 MFI, p < 0.01), from KIR2DL5^+^ donors produced significantly higher levels of icNO compared to KIR2DL5^-^ donors. Similarly, after BCG stimulation, CD4^+^ (296.6 ± 62.4 vs. 115.9 ± 23.8 MFI, p < 0.05) and CD8^+^ (385.6 ± 68.3 vs. 148.4 ± 22.8 MFI, p < 0.01) T cells, as well as NK cells (278.7 ± 53.4 vs. 113.5 ± 25.2 MFI, p < 0.05), from KIR2DL5^+^ donors exhibited significantly higher icNO production than KIR2DL5^-^ donors (Figure 5).

### 3.6 KIR2DL5 is predominantly associated with small (<3 cm), solid-pattern NMIBC in non-smoking patients

**FIGURE 6:**
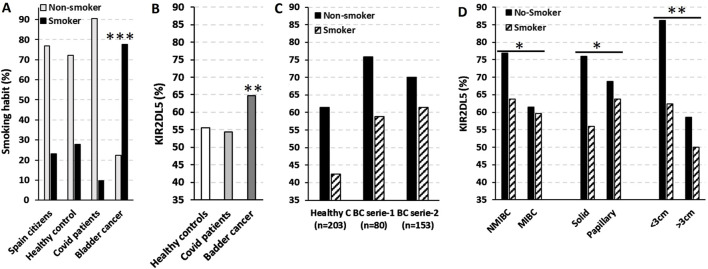
KIR2DL5 is predominantly associated with small (<3 cm), solid-pattern NMIBC in non-smoking patients. **(A)** Smoking prevalence in the general Spanish population (data from https://www.ine.es/infografias/infografia_tabaco.pdf), healthy controls (HC, n = 201), COVID-19 patients (n = 458) (Niño-Ramírez et al., 2024), and bladder cancer patients (BC, n = 263). **(B)** Frequency of KIR2DL5 in HC, COVID-19, and BC patients. **(C)** Smoking distribution in HC (n = 203) and two independent BC cohorts (series 1, n = 80; series 2, n = 153). **(D)** Frequency of KIR2DL5 in BC patients, stratified by tumor stage, pattern, and size. *, p < 0.05; **, p < 0.01; ***, p < 0.001 based on chi-square tests.

The strong association between smoking and BC (Freedman, 2011; Halaseh et al., 2022) was confirmed in our cohort, where smoking was present in 77.7% of BC patients (p < 0.001), compared to only 23.0%, 27.9%, and 9.6% in the general Spanish population, HC from our study, and COVID-19 patients from the confirmatory series, respectively (Figure 6A).

As shown in Figure 6B, the frequency of KIR2DL5 was significantly higher in BC patients (64.7%, p < 0.01) compared to HC (55.5%) and COVID-19 patients (54.4%) from the confirmatory series. Interestingly, KIR2DL5 frequency was higher among non-smoking HC (61.4% vs. 42.5%) and BC patients from series-1 (75.9% vs. 58.8%) and series-2 (70.0% vs. 51.4%) compared to smokers (Figure 6C). Furthermore, non-smoking BC patients exhibited higher KIR2DL5 frequencies in NMIBC compared to MIBC cases (76.9% vs. 63.7%, p < 0.05), in solid-pattern tumors compared to papillary tumors (76.0% vs. 56.0%, p < 0.05), and in tumors <3 cm compared to those >3 cm in size (86.2% vs. 62.4%, p < 0.01) (Figure 6D).

## 4 Discussion

According to the American Cancer Society, smokers are at least three times more likely to develop BC compared to non-smokers. In fact, tobacco use is implicated in approximately 50% of male cases and 20%–30% of female cases. Carcinogens in tobacco smoke, particularly aromatic amines like 2-naphthylamine and 4-aminobiphenyl, enter the bloodstream, are filtered by the kidneys, and accumulate in urine for prolonged periods, inducing DNA damage through adduct formation and mutagenic processes in bladder cells (Freedman, 2011; Halaseh et al., 2022). Additionally, exposure to harmful chemicals (such as aromatic amines and benzidine, which are responsible for 5%–10% of BC), previous cancer treatments, chronic bladder inflammation, *Schistosomiasis haematobium* infection, obesity, diet, gender, age, or a family history of BC can also be predisposing factors (Halaseh et al., 2022). However, studies have failed to recognize important germline genetic factors. Genome-wide studies (Gu and Wu, 2011) have identified a small correlation with a genetic predisposition to BC for N-acetyltransferase 2 (NAT2) and deletion of glutathione S-transferase (GSTM1) genes, both connected with the ability to metabolize aromatic amines and therefore related to the environmental carcinogen exposure and cigarette smoking (García-Closas et al., 2005). Increased incidence of BC has also been related to mutations in the tumor-suppressor gene phosphatase and tensin homolog (PTEN) and the DNA mismatch repair gene MutS homolog 2 (MSH2), which are seen in Cowden and Lynch syndromes, respectively (Riegert-Johnson et al., 2010; van der Post et al., 2010). The absence of a clear Mendelian inheritance pattern of BC suggests that not a monogenic system, but a multigenic/polymorphic loci might be involved. Our data demonstrate a specific and significant association between KIR2DL5 and elevated BC risk across the entire cohort and within both independent patient series included in the study. This association is further emphasized by the heightened KIR2DL5 frequency observed in non-smoking patients, whose reduced tobacco exposure likely minimizes masking of KIR2DL5’s inherent predisposing effects. The findings suggest a distinct biological role for KIR2DL5 in bladder carcinogenesis, independent of smoking-related pathways.

The functional interpretation of KIR2DL5 has been complicated by its strong linkage disequilibrium with other aKIRs in the haplotype B. Prior studies have primarily associated haplotype B or collective aKIR clusters -rather than KIR2DL5 itself-with diverse clinical outcomes (Sullivan et al., 2014; Nowak et al., 2015; Pamuk et al., 2015; Misra et al., 2016; Barani et al., 2018; Hernandez et al., 2018). In contrast, our findings demonstrate a significant and specific association between KIR2DL5 and the risk of BC, independent of confounding variables such as coexisting aKIRs, sex, age, or tobacco smoking. Moreover, although the presence of KIR2DL5 significantly influences the presence of circulating NK cells differentially expressing KIR2DS1, KIR2DL2, and KIR3DL1, these cells appear to be more closely associated with the development of cancers in general, rather than with BC in particular. This highlights KIR2DL5 as a distinct immunogenetic factor in bladder carcinogenesis, warranting mechanistic studies to dissect its role in tumor-immune interactions. However, neither KIR2DL5 nor the presence of aKIRs within the B-haplotype (data not shown) demonstrated prognostic relevance for BC outcomes following treatment initiation. This suggests that while KIR2DL5 may influence BC predisposition, it does not appear to modulate therapeutic response or disease progression in treated patients. Unfortunately, the genotyping method used in our study did not allow us to specifically distinguish whether this association was attributable to the KIR2DL5A or KIR2DL5B paralog. However, since only KIR2DL5A*001 is known to be expressed on the cell membrane (Cisneros et al., 2012), its interaction with its PVR ligands indicates that this KIR2DL5 allotype may be responsible for the predisposition to breast cancer.

The absence of prior evidence for KIR2DL5’s distinct role in BC pathophysiology complicates direct comparison with our findings. To address this issue, functional assays were conducted to characterize tumor-associated immune effector mechanisms, aiming to identify specific immunological pathways modulated by KIR2DL5. KIR2DL5 showed no association with altered T cell (CD4^+^ and CD8^+^) or NK cell proliferation, cytokine production, or cytotoxicity. However, it correlated with elevated icNO following *in vitro* stimulation with anti-CD3/CD28 or BCG in both immune cell types. Although the sample size of these exploratory assays may have been insufficient to detect subtle modulations in cytotoxicity, proliferation, or cytokine secretion, it proved sufficiently robust to identify a significant increase in icNO in KIR2DL5+ donors. These findings position dysregulation of the NO pathway as the most relevant biological mechanism explaining the genetic association reported in this manuscript. Therefore, the interaction of KIR2DL5 with its PVR ligand on transformed or infected cells could initiate SHP-2–dependent signaling (Yusa et al., 2002; Yusa and Campbell, 2003; Ren et al., 2022), resulting in heightened icNO production during NK and/or T cell immune activation, which may have direct pathological consequences. Although SHIP2 is not directly involved in NO production, it is involved in regulating the activity of NADPH oxidases (NOX), which are enzymes that produce reactive oxygen species. Some studies suggest that NOX enzymes can also affect NO bioavailability, potentially through reactions with NO or by influencing the activity of nitric oxide synthase (iNOS). Therefore, SHIP2’s influence on NOX activity could indirectly affect NO levels (Watt et al., 2017). Thus, the excess NO produced during immune responses could diffuse into the circulation and be metabolized into nitrate, a stable urinary excretory product that accumulates in the bladder (Stichtenoth et al., 1995; SEVER et al., 2008). This nitrate reservoir could subsequently contribute to bladder carcinogenesis through localized genotoxic or inflammatory mechanisms (Chiu et al., 2007; Espejo-Herrera et al., 2015; Jones et al., 2016; Barry et al., 2020). Further studies are needed to test our hypothesis by analyzing NO production and its associated molecular pathways in NK and T cells isolated from the tumor infiltrate, in order to gain deeper insight into the role of the tumor microenvironment.

Furthermore, the potential direct involvement of NO in modulating NK and T cell antitumor activity warrants consideration. While NO does not significantly influence the effector phase of cytokine-stimulated NK cell cytotoxicity, elevated concentrations during the afferent phase -particularly under IL-12 and TNFα priming-may attenuate NK lytic capacity (Salvucci et al., 1998). Notably, tumor-infiltrating NK cells expressing the inhibitory receptor KIR2DL5 are frequently observed in malignancies with high PVR expression (Ren et al., 2022). Our findings imply these cells may exhibit enhanced NO production, which could subsequently upregulate PVR expression via feedback mechanisms analogous to those documented in myeloma models (Fionda et al., 2015). As PVR engages inhibitory immune checkpoints (CD96, TIGIT, KIR2DL5), this NO-PVR axis may establish a self-reinforcing loop that suppresses effector lymphocyte function, thereby amplifying immune evasion (Kučan Brlić et al., 2019). The feasibility of targeting this inhibitory loop in conjunction with other protein-targeted immunotherapies in BC is supported by comprehensive proteomic analyses that have systematically mapped the landscape of druggable targets within this malignancy (Mertins et al., 2016).

Tobacco smoking is known to impair endothelial NO synthesis (Toda and Toda, 2010), prompting investigation into whether it could also suppress NO production by immune cells. This mechanism may explain the higher prevalence of KIR2DL5 observed in BC patients who do not smoke in our cohort, suggesting that the strong carcinogenic effects of tobacco could mask this association. Furthermore, the inverse relationship we observed between smoking, commonly linked to MIBC and KIR2DL5, which is associated with small, solid-pattern NMIBC, supports this hypothesis.

However, this study leaves unresolved questions: what molecular pathways underlie the potential mechanistic link between KIR2DL5 and NO overproduction? and how do smoking-induced epigenetic modifications interact with this immunoregulatory axis? These limitations underscore the need for multicenter collaborations to validate these associations in expanded cohorts and investigate potential confounding from unmeasured inflammatory mediators, while integrating multi-omics approaches to elucidate NO-KIR2DL5 crosstalk.

## 5 Conclusion

Although these findings require confirmation in larger cohorts, our data suggest that KIR2DL5 may be associated with bladder cancer risk independently of age, sex, and tobacco smoking exposure. The immunological mechanisms underlying this association remain poorly understood; however, preliminary evidence indicates that dysregulated NO production by immune effector cells could play a key role in mediating this relationship. Future studies are needed to unravel the interactions between KIR2DL5-expressing lymphocytes and NO signaling pathways within the bladder tumor microenvironment.

## Data Availability

The raw data supporting the conclusions of this article will be made available by the authors, without undue reservation.

## References

[B1] Al-Zubi M. T. Demour S.A. Al-Rawashdah S. F. Carbone A. Pastore A. L. Abuhamad M. (2022). Does post-void residual urine volume affect potential recurrence risk for non-muscle invasive bladder cancer? Future Sci. OA 8. 10.2144/fsoa-2022-0045 36788983 PMC9912276

[B2] Barani S. Khademi B. Ashouri E. Ghaderi A. (2018). KIR2DS1, 2DS5, 3DS1 and KIR2DL5 are associated with the risk of head and neck squamous cell carcinoma in Iranians. Hum. Immunol. 79, 218–223. 10.1016/j.humimm.2018.01.012 29408295

[B3] Barry K. H. Jones R. R. Cantor K. P. Beane Freeman L. E. Wheeler D. C. Baris D. (2020). Ingested nitrate and nitrite and bladder cancer in northern new England. Epidemiology 31, 136–144. 10.1097/EDE.0000000000001112 31577632 PMC6927574

[B4] Bogdan C. (2001). Nitric oxide and the immune response. Nat. Immunol. 2, 907–916. 10.1038/ni1001-907 11577346

[B5] Chiu H.-F. Tsai S.-S. Yang C.-Y. (2007). Nitrate in drinking water and risk of death from bladder cancer: an ecological case-control study in Taiwan. J. Toxicol. Environ. Health A 70, 1000–1004. 10.1080/15287390601171801 17497410

[B6] Cisneros E. Moraru M. Gómez-Lozano N. López-Botet M. Vilches C. (2012). KIR2DL5: an orphan inhibitory receptor displaying complex patterns of polymorphism and expression. Front. Immunol. 3, 289. 10.3389/fimmu.2012.00289 23060877 PMC3443818

[B7] Espejo-Herrera N. Cantor K. P. Malats N. Silverman D. T. Tardón A. García-Closas R. (2015). Nitrate in drinking water and bladder cancer risk in Spain. Environ. Res. 137, 299–307. 10.1016/j.envres.2014.10.034 25601732

[B8] Fionda C. Abruzzese M. P. Zingoni A. Soriani A. Ricci B. Molfetta R. (2015). Nitric oxide donors increase PVR/CD155 DNAM-1 ligand expression in multiple myeloma cells: role of DNA damage response activation. BMC Cancer 15, 17. 10.1186/s12885-015-1023-5 25609078 PMC4311457

[B9] Freedman N. D. Silverman D. T. Hollenbeck A. R. Schatzkin A. Abnet C. C. (2011). Association between smoking and risk of bladder cancer among men and women. JAMA 306, 737–745. 10.1001/jama.2011.1142 21846855 PMC3441175

[B10] García-Closas M. Malats N. Silverman D. Dosemeci M. Kogevinas M. Hein D. W. (2005). NAT2 slow acetylation, GSTM1 null genotype, and risk of bladder cancer: results from the Spanish bladder cancer study and meta-analyses. Lancet 366, 649–659. 10.1016/S0140-6736(05)67137-1 16112301 PMC1459966

[B11] Gimeno L. González-Lozano I. Soto-Ramírez M. F. Martínez-Sánchez M. V. López-Cubillana P. Fuster J. L. (2021). CD8+ T lymphocytes are sensitive to NKG2A/HLA-E licensing interaction: role in the survival of cancer patients. Oncoimmunology 10, 1986943. 10.1080/2162402X.2021.1986943 34676148 PMC8525952

[B12] Gómez-Lozano N. Gardiner C. Parham P. Vilches C. (2002). Some human KIR haplotypes contain two KIR2DL5 genes: KIR2DL5A and KIR2DL5B. Immunogenetics 54, 314–319. 10.1007/s00251-002-0476-2 12185535

[B13] Gu J. Wu X. (2011). Genetic susceptibility to bladder cancer risk and outcome. Per Med. 8, 365–374. 10.2217/pme.11.15 21927616 PMC3172962

[B14] Guillamón C. F. Martínez-Sánchez M. V. Gimeno L. Mrowiec A. Martínez-García J. Server-Pastor G. (2018). NK cell education in tumor immune surveillance: DNAM-1/KIR receptor ratios as predictive biomarkers for solid tumor outcome. Cancer Immunol. Res. 6, 1537–1547. 10.1158/2326-6066.CIR-18-0022 30242020

[B15] Guillamón C. F. Gimeno L. Server G. Martínez-Sánchez M. V. Escudero J. F. López-Cubillana P. (2021). Immunological risk stratification of bladder cancer based on peripheral blood natural killer cell biomarkers. Eur. Urol. Oncol. 4, 246–255. 10.1016/j.euo.2019.04.009 31411976

[B16] Halaseh S. A. Halaseh S. Alali Y. Ashour M. E. Alharayzah M. J. (2022). A review of the etiology and epidemiology of bladder cancer: all you need to know. Cureus 14, e27330. 10.7759/cureus.27330 36042998 PMC9411696

[B17] Hernandez E. G. Partida-Rodriguez O. Camorlinga-Ponce M. Nieves-Ramirez M. Ramos-Vega I. Torres J. (2018). Genotype B of killer cell immunoglobulin-like receptor is related with gastric cancer lesions. Sci. Rep. 8, 6104–6109. 10.1038/s41598-018-24464-2 29666399 PMC5904182

[B18] Hsu K. C. Chida S. Geraghty D. E. Dupont B. (2002). The killer cell immunoglobulin-like receptor (KIR) genomic region: gene-order, haplotypes and allelic polymorphism. Immunol. Rev. 190, 40–52. 10.1034/j.1600-065x.2002.19004.x 12493005

[B19] Husain B. Ramani S. R. Chiang E. Lehoux I. Paduchuri S. Arena T. A. (2019). A platform for extracellular interactome discovery identifies novel functional binding partners for the immune receptors B7-H3/CD276 and PVR/CD155. Mol. and Cell. Proteomics 18, 2310–2323. 10.1074/mcp.TIR119.001433 31308249 PMC6823854

[B20] Jones R. R. Weyer P. J. DellaValle C. T. Inoue-Choi M. Anderson K. E. Cantor K. P. (2016). Nitrate from drinking water and diet and bladder cancer among postmenopausal women in Iowa. Environ. Health Perspect. 124, 1751–1758. 10.1289/EHP191 27258851 PMC5089883

[B21] Kučan Brlić P. Lenac Roviš T. Cinamon G. Tsukerman P. Mandelboim O. Jonjić S. (2019). Targeting PVR (CD155) and its receptors in anti-tumor therapy. Cell Mol. Immunol. 16, 40–52. 10.1038/s41423-018-0168-y 30275538 PMC6318332

[B22] Mertins P. Mani D. R. Ruggles K. V. Gillette M. A. Clauser K. R. Wang P. (2016). Proteogenomics connects somatic mutations to signalling in breast cancer. Nature 534, 55–62. 10.1038/nature18003 27251275 PMC5102256

[B23] Misra M. K. Prakash S. Moulik N. R. Kumar A. Agrawal S. (2016). Genetic associations of killer immunoglobulin like receptors and class I human leukocyte antigens on childhood acute lymphoblastic leukemia among north Indians. Hum. Immunol. 77, 41–46. 10.1016/j.humimm.2015.10.009 26472014

[B24] Moch H. Humphrey P. A. Ulbright T. M. (2016). WHO classification of tumours of the urinary system and Male genital organs. Fourth edition. 10.1016/j.eururo.2016.02.028 26996659

[B25] Moretta L. Moretta A. (2004). Killer immunoglobulin-like receptors. Curr. Opin. Immunol. 16, 626–633. 10.1016/j.coi.2004.07.010 15342010

[B26] Muñoz Resta I. Bedrina B. Martínez-Planes E. Minguela A. Galindo F. (2021). Detection of subcellular nitric oxide in mitochondria using a pyrylium probe: assays in cell cultures and peripheral blood. J. Mater Chem. B 9, 9885–9892. 10.1039/d1tb02326h 34821904

[B27] Niño-Ramírez J. E. Alcoceba M. Gutiérrez-Zufiaurre M. N. Marcos M. Gil-Etayo F. J. Bartol-Sánchez M. R. (2024). Killer-cell immunoglobulin-like receptor polymorphism is associated with COVID-19 outcome: results of a pilot observational study. HLA 104, e15640. 10.1111/tan.15640 39148254

[B28] Nooshinfar E. Bashash D. Abbasalizadeh M. Safaroghli-Azar A. Sadreazami P. Esmaeil Akbari M. (2017). The molecular mechanisms of tobacco in cancer pathogenesis. Iran. J. Cancer Prev. 10.17795/ijcp-7902

[B29] Nowak J. Kościńska K. Mika-Witkowska R. Rogatko-Koroś M. Mizia S. Jaskuła E. (2015). Role of donor activating KIR-HLA ligand-mediated NK cell education status in control of malignancy in hematopoietic cell transplant recipients. Biol. Blood Marrow Transpl. 21, 829–839. 10.1016/j.bbmt.2015.01.018 25617806

[B30] Ordóñez D. Meenagh A. Gómez-Lozano N. Castaño J. Middleton D. Vilches C. (2008). Duplication, mutation and recombination of the human orphan gene KIR2DS3 contribute to the diversity of KIR haplotypes. Genes Immun. 9, 431–437. 10.1038/gene.2008.34 18480828

[B31] Pamuk G. E. Tozkir H. Uyanik M. S. Gurkan H. Duymaz J. Pamuk O. N. (2015). Natural killer cell killer immunoglobulin-like gene receptor polymorphisms in non-Hodgkin lymphoma: possible association with clinical course. Leuk. Lymphoma 56, 2902–2907. 10.3109/10428194.2015.1014361 25699652

[B32] Ren X. Peng M. Xing P. Wei Y. Galbo P. M. Corrigan D. (2022). Blockade of the immunosuppressive KIR2DL5/PVR pathway elicits potent human NK cell–mediated antitumor immunity. J. Clin. Investigation 132, e163620. 10.1172/JCI163620 36377656 PMC9663162

[B33] Riegert-Johnson D. L. Gleeson F. C. Roberts M. Tholen K. Youngborg L. Bullock M. (2010). Cancer and Lhermitte-Duclos disease are common in Cowden syndrome patients. Hered. Cancer Clin. Pract. 8, 6. 10.1186/1897-4287-8-6 20565722 PMC2904729

[B34] Ruiz-Lorente I. Gimeno L. López-Abad A. López Cubillana P. Fernández Aparicio T. Asensio Egea L. J. (2025). Differential role of NKG2A/HLA-E interaction in the outcomes of bladder cancer patients treated with M. bovis BCG or other therapies. Biomedicines 13, 156. 10.3390/biomedicines13010156 39857739 PMC11760850

[B35] Salvucci O. Kolb J. P. Dugas B. Dugas N. Chouaib S. (1998). The induction of nitric oxide by Interleukin-12 and tumor necrosis Factor in HumanhNaturnl Killek Cellsc Relatronship With whe Regulrtion of LyticlActivaty. Blood 92, 2093–2102. 10.1182/blood.V92.6.2093 9731067

[B36] Sever R. Cook T. Cattell V. (2008). Urinary excretion of nitrite and nitrate in experimental glomerulonephritis reflects systemic immune activation and not glomerular synthesis. Clin. Exp. Immunol. 90, 326–329. 10.1111/j.1365-2249.1992.tb07950.x 1424292 PMC1554600

[B37] Siemens D. R. Hu N. Sheikhi A. K. Chung E. Frederiksen L. J. Pross H. (2008). Hypoxia increases tumor cell shedding of MHC class I chain-related molecule: role of nitric oxide. Cancer Res. 68, 4746–4753. 10.1158/0008-5472.CAN-08-0054 18559521

[B38] Stichtenoth D. O. Fauler J. Zeidler H. Frolich J. C. (1995). Urinary nitrate excretion is increased in patients with rheumatoid arthritis and reduced by prednisolone. Ann. Rheum. Dis. 54, 820–824. 10.1136/ard.54.10.820 7492221 PMC1010017

[B39] Sullivan E. M. Jeha S. Kang G. Cheng C. Rooney B. Holladay M. (2014). NK cell genotype and phenotype at diagnosis of acute lymphoblastic leukemia correlate with postinduction residual disease. Clin. Cancer Res. 20, 5986–5994. 10.1158/1078-0432.CCR-14-0479 25281696 PMC4252745

[B40] Toda N. Toda H. (2010). Nitric oxide-mediated blood flow regulation as affected by smoking and nicotine. Eur. J. Pharmacol. 649, 1–13. 10.1016/j.ejphar.2010.09.042 20868673

[B41] van der Post R. S. Kiemeney L. A. Ligtenberg M. J. L. Witjes J. A. Hulsbergen-van de Kaa C. A. Bodmer D. (2010). Risk of urothelial bladder cancer in Lynch syndrome is increased, in particular among MSH2 mutation carriers. J. Med. Genet. 47, 464–470. 10.1136/jmg.2010.076992 20591884 PMC2991077

[B42] Vilches C. Rajalingam R. Uhrberg M. Gardiner C. M. Young N. T. Parham P. (2000). KIR2DL5, a novel killer-cell receptor with a D0-D2 configuration of Ig-Like domains. J. Immunol. 164, 5797–5804. 10.4049/jimmunol.164.11.5797 10820258

[B43] Watt N. T. Gage M. C. Patel P. A. Viswambharan H. Sukumar P. Galloway S. (2017). Endothelial SHIP2 suppresses Nox2 NADPH oxidase-dependent vascular oxidative stress, endothelial dysfunction, and systemic insulin resistance. Diabetes 66, 2808–2821. 10.2337/db17-0062 28830894

[B44] Yeo J. Ko M. Lee D.-H. Park Y. Jin H.-S. (2021). TIGIT/CD226 axis regulates anti-tumor immunity. Pharm. (Basel) 14, 200. 10.3390/ph14030200 33670993 PMC7997242

[B45] Yusa S. Campbell K. S. (2003). Src homology region 2-Containing protein tyrosine Phosphatase-2 (SHP-2) can play a direct role in the inhibitory function of killer cell Ig-Like receptors in human NK cells. J. Immunol. 170, 4539–4547. 10.4049/jimmunol.170.9.4539 12707331

[B46] Yusa S. Catina T. L. Campbell K. S. (2002). SHP-1- and phosphotyrosine-independent inhibitory signaling by a killer cell Ig-like receptor cytoplasmic domain in human NK cells. J. Immunol. 168, 5047–5057. 10.4049/jimmunol.168.10.5047 11994457

[B47] Zhang J. Zhu Y. Wang Q. Kong Y. Sheng H. Guo J. (2020). Poliovirus receptor CD155 is up-regulated in muscle-invasive bladder cancer and predicts poor prognosis. Urol. Oncol. 38, 41.e11–e41. 10.1016/j.urolonc.2019.07.006 31383549

